# Ferulic acid in synergy with retinol alleviates oxidative injury of HaCaT cells during UVB-induced photoaging

**DOI:** 10.18632/aging.205749

**Published:** 2024-04-18

**Authors:** Peng Shu, Jiaxin Mo, Zunjiang Li, Mingjie Li, Wei Zhu, Zhiyun Du

**Affiliations:** 1 State Key Laboratory Basis of Xinjiang Indigenous Medicinal Plants Resource Utilization, CAS Key Laboratory of Chemistry of Plant Resources in Arid Regions, Xinjiang Technical Institute of Physics and Chemistry, Chinese Academy of Sciences, Urumqi 830011, Xinjiang, P.R. China; 2 The Second Clinical College, Guangzhou University of Chinese Medicine, Guangzhou 510006, Guangdong, P.R. China; 3 HBN Research Institute and Biological Laboratory, Shenzhen Hujia Technology Co., Ltd., Shenzhen 518000, Guangdong, P.R. China; 4 University of Chinese Academy of Sciences, Beijing 100049, P.R. China; 5 Guangdong Provincial Key Laboratory of Clinical Research on Traditional Chinese Medicine Syndrome, Guangzhou 510006, Guangdong, P.R. China

**Keywords:** retinol, ferulic acid, cooperative photoprotection, skin aging, oxidative stress

## Abstract

Application of retinol (Vitamin A, VA) in skincare is limited for instability, poor water solubility, and skin intolerance that combats skin aging. We employed computer-aided virtual screening and cell experiments with transcriptomics, thereby unveiling the comprehensive gene expression and regulation pathway of photoaging HaCaT cell treated with ferulic acid (FA) in synergizing with VA. Through network pharmacology analysis, the combined use of VA and FA exhibited highly correlated cross-targets with skin aging acting on EGFR, PTPN1, ESR2, GSK3B, BACE1, PYGL, PTGS2 and APP. The indicators of oxidative stress, such as SOD, GSH, MDA, CAT and ROS in HaCaT cells after co-administration, were significantly improved from those in photoaging group (*p*<0.0001). 155 differential expressed genes (DEGs) were specific between groups, while reducing the expression of PTGS2 was identified as an important regulatory factor in photoaging HaCaT cells by VA and FA. Those DEGs of co-administration group focused on oxidative-reduction enzyme activity, skin growth, keratinization, and steroid biosynthesis. Apparently, the co-administration of VA and FA effectively mitigated the process of UVB-induced photoaging by reducing oxidative stress injury, inflammation responses, and regulating cell growth. This synergistic approach significantly slowed down the photoaging progression and improved the applied performance of VA in HaCaT cells.

## INTRODUCTION

Aging of the skin is an intricate and unchangeable biological process. UVR, or ultraviolet light radiation, can cause significant harm to skin from the sun and artificial sources. This process speeds up skin aging, called photoaging [1]. Based on wavelength, UVR can be divided into three groups: UVA (320–400 nm), UVB (280–320 nm), and UVC (100–280 nm). UVR have detrimental effects that are either short-term or long-term, including acute erythema, wrinkles, pigmentation, and the breakdown of collagen and elastin [2]. UVB radiation can penetrate the epidermis of the skin and cause function damage to cells [3]. Age and prolonged UVB exposure inevitably cause wrinkles, pigmentation, laxis of the epidermis, and a decrease in dermal and epidermal thickness. DNA damage, oxidative stress, cellular inflammatory responses, and microcirculation alterations all contribute to the development of photoaging [4]. Reactive oxygen species (ROS) are produced and accumulate during the photoaging process, mostly for cell peroxidation and free radicals. Hence, interfere with the body’s dynamic scavenging of such metabolites, disturbing the skin’s regular processes [5, 6]. ROS production could activate the signaling pathway phosphatidylinositol 3-kinase (PI3K)/Akt and mitogen-activated protein kinases (MAPKs), which caused upregulated transcription of inflammatory mediators [7]. Prostaglandin Endoperoxide Synthase (PTGS2) causes continuous damage in proliferation and dermoepidermal junction of skin later. Cyclooxygenase-2 (COX-2) is encoded by the transcription of the PTGS2 gene, take part in produce of ROS and several inflammatory cytokines in skin such as IL-1. UVR activates the NFAT, NF-κB pathway to increase COX-2 transcription by stimulating ROS produced in cells [8]. The antioxidant defense mechanism of the skin is interfered with by ROS, which in turn induces inflammation-induced aging due to overexpression of COX-2 [9]. The best defense against premature skin aging is to stay out of the UVR, especially as UVB might have an impact.

Retinol (Vitamin A, VA) has been shown to have both curative and preventive benefits on the aging process of the skin. VA restored dermal thickness of skin *ex vivo* and *in vivo* by effectively blocking the reversion of elastic fiber deposition and organization [10]. Through randomized, double-blind clinical trial during 12 weeks, 0.5% VA cream applied on full face for participants ages 31-56 twice a day, could reduce the wrinkle surface area and pigmentation significantly [11]. However, there was restricted application associated with high doses of VA, including sensitivity, stinging, burning, cutaneous erythema, peeling, and pruritus [12]. Treatment effect of skin aging with VA is dose-dependent, and there is often no significant improvement in photoaging skin at low doses, but high concentrations of 0.5%~1% VA might cause more frequent and intense symptoms such as dermatitis reaction [13, 14]. Efforts have been made to reduce the irritation of VA while preserving the efficacy. In cosmeceuticals, combining with different drugs, Chien verified that cosmetic product with 1% VA, 0.05% retinyl acetate, 0.05% retinol palmitate moderated facial photodamage persons or the more serious. Patients only treated with VA experienced tropical skin irritation, which is 6 times more frequently than those who developed intolerance with combinations [12]. Therefore, combining other substances may synergistically take full advantage of VA.

For example, as a commonly used antioxidant in skin care products, ferulic acid (FA) has a very strong ability in scavenging free radicals, improving cellular antioxidant defense system and inhibiting oxidative stress damage to achieve cell protection, which is often used in combination with other ingredients in skin care products [15]. Relevant literature studies have shown that the different ratios of antioxidants companied with anti-aging ingredients can reduce the intolerance to cells. For instance, topical application of combining vitamin C, vitamin E (VE), and FA for 2 weeks decreased the melanin index between 26-53 years old men and women [16]. However, the combination work of VA and FA are unexplored. How to efficiently obtain the matching ingredients and dosages that reduce the irritation of retinol is crucial.

Currently, the idea that organs should be the focus of medical treatment is counterproductive to medication discovery and research. Preclinical animal models mostly mimic the symptoms of illnesses, with less understanding of the underlying causes of illness [17]. For example, ACE inhibitors, such as sacubitril/valsartan, are used for heart failure [18]. But this treatment is only focus on the symptoms, rather than the cause of the disease. As consequently, the technique known as network pharmacology is introduced using virtual computing, high-throughput data processing, and the creation of bioinformatics networks [19]. Network pharmacology describes the occurrence of disease and the possibility of treatment through endotypes defined by causality [20], which aims at specific proteins in disease developing pathways directly or indirectly. It interprets the comorbidities of disease phenotypes through multi-target signaling pathways, accurately intervenes in each process of disease occurrence, and accelerates clinical translation [21]. Network pharmacology maximum save times and pinpoints the effective drugs for diseases from thousands of compounds. The theories and strategies of network pharmacology have been successfully applied to the analysis of single herbs and prescriptions in traditional Chinese medicine. For example, Moluodan in the treatment of chronic atrophic gastritis which verified by GSE-1 cells [22]; Based on the metabolic profile of Alismatis rhizoma in mice, biomarkers against hyperlipidemia were identified by compound-metabolite network, which verified by qPCR [23].

The main objective of this project is to promote the synergistic protection for photoaging and reduce the irritation of VA by formulating a mixture with FA. To create a photoaging model that would show the combined protective influence of VA and FA on UVB-damaged HaCaT cells. Applied with network pharmacology, excluded the potential cooperative medication for the VA. The non-linear mixed amount with zero amount (NLMAZ) was used to modeling synergism patterns administrated with various of ratios of FA and VA [24]. Then, observed the morphological changes of HaCaT cells, detected oxidative stress-related inflammatory factors such as superoxide dismutase (SOD), malondialdehyde (MDA), reduced glutathione (GSH), catalase (CAT) and reactive oxygen species (ROS) indicators. Transcriptomics was used to verify and find the key regulatory targets of VA and FA on UVB-induced photoaging HaCaT cells. To confirm the profound regulatory connection that exists between its transcription and the level of keratinocyte senescence, the vital regulatory protein COX-2 was analyzed.

## RESULTS

### Collecting targets of retinol, ferulic acid and skin aging

According to the TCMSP, Drugbank and SwissTargetPrediction Database, 134 targets of retinol (Supplementary Table 1) and 107 targets of ferulic acid (Supplementary Table 2) were collected. A total of 774 skin aging targets were also obtained from GWAS database and GeneCards database after deduplication (Supplementary Table 3).

### Identification synergic biological function of VA and FA

When conducting Venn analysis on synergic targets acting on skin aging of VA and FA, 82 and 68 genes were intersected by VA and FA at the target to skin aging (Supplementary Table 4), respectively. Enrichment analysis showed that 45 and 31 in these targets were enriched in re-inflammatory processes, antioxidant function and regulation of immune function. Those genes were considered as the common targets of VA and FA for skin aging diseases.

**Figure 1:**
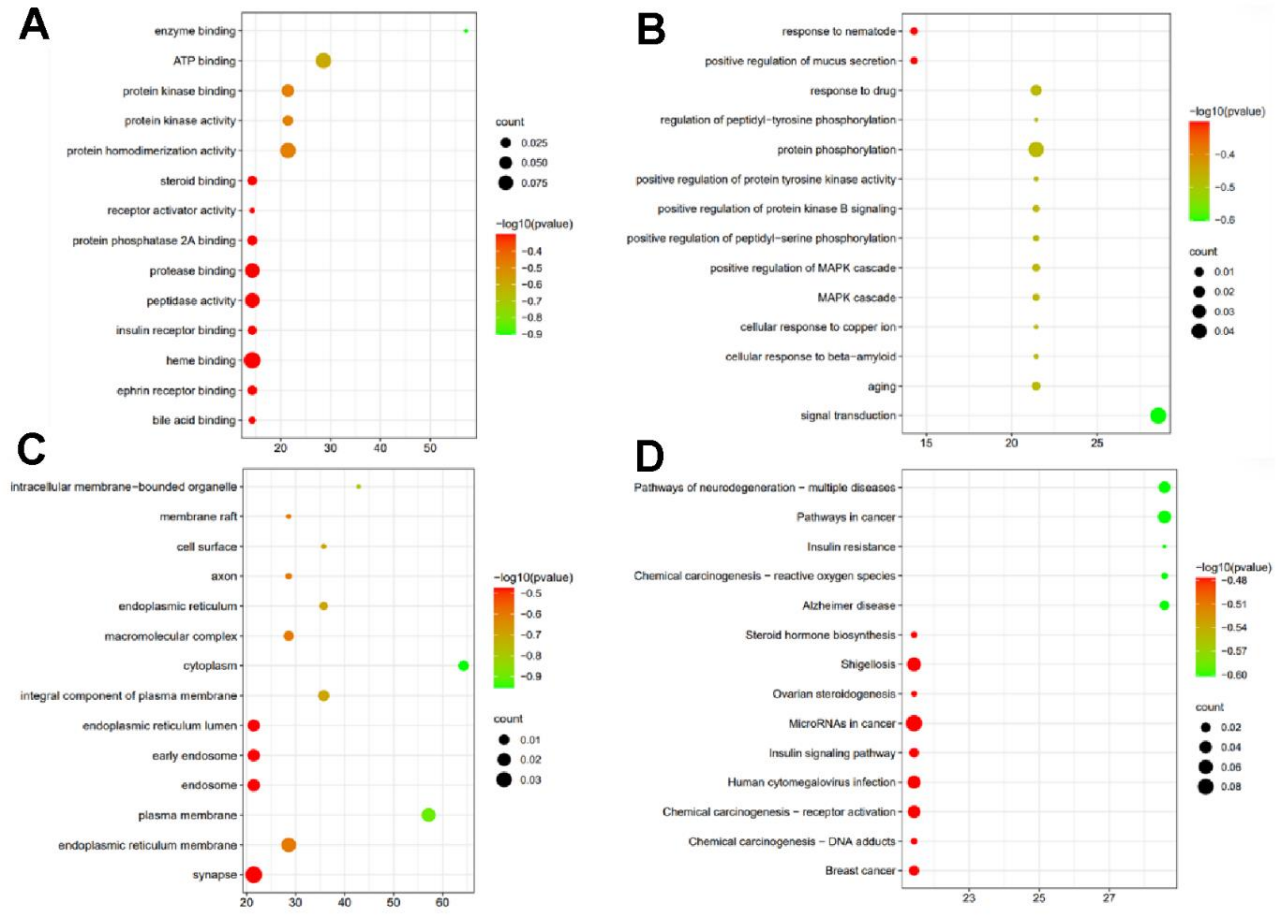
**Synergic biological function of VA and FA.** (**A**) Synergic targets of VA and FA enriched in biological processes (MF); (**B**) Synergic targets of VA and FA enriched in cellular component (BP); (**C**) Synergic targets of VA and FA enriched in molecular function (CC); (**D**) Synergic targets of VA and FA enriched in KEGG enrichment of the intersection targets between SGR predicted targets and heart failure.

The results showed that VA and FA were cooperative in regulating cell proliferation, oxidation–reduction process and apoptotic process, etc. (Figure 1B). At molecular level, VA and FA mainly acted on enzyme binding, receptor activator activity, protein kinase binding, ATP binding (Figure 1A) in the intracellular membrane-bounded organelle, membrane raft, cell surface, endoplasmic reticulum, plasma membrane, endoplasmic reticulum membrane and mitochondrion etc. (Figure 1C). In terms of its involved pathways, it showed that synergic biological function of VA and FA may be effective via regulating MAPK signaling pathway, calcium signaling pathway, cardiac muscle contraction, etc. (Figure 1D).

### Identification of DEGs in skin aging

**Figure 2:**
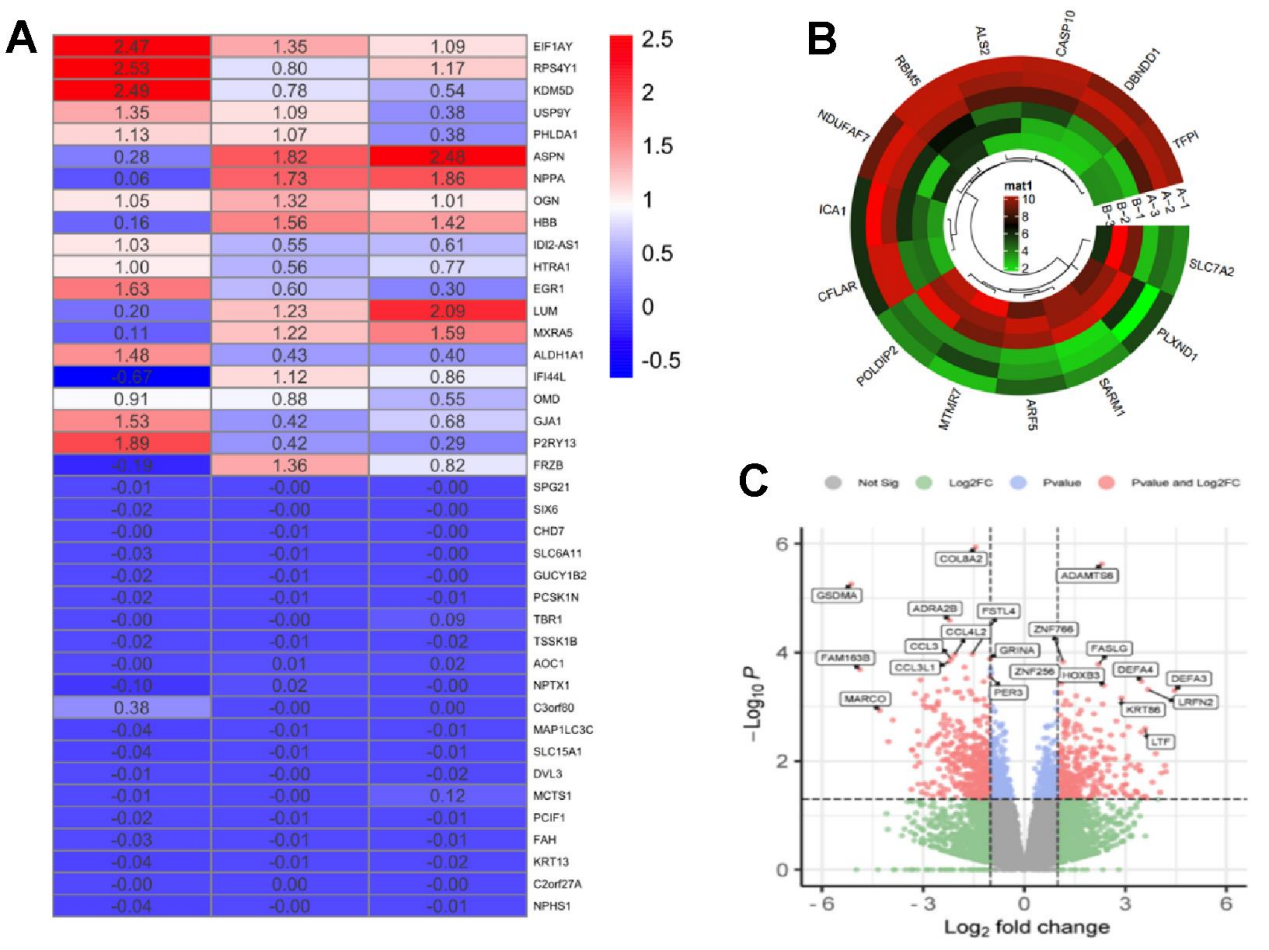
**Differentially expressed genes of skin aging identification.** (**A**) Heat map of DEGs in the GSE155789; (**B**) Cluster of DEGs in the GSE155789; (**C**) Volcano plot of differentially DEGs in the GSE155789. group C: normal HaCaT cells; group VA: 100 nM retinol in HaCaT cells.

We identified 252 DEGs in the GSE155789 dataset using the GEO2R analysis in GEO database (Supplementary Table 5). Based on the analysis, 72 genes were up-regulated and 179 genes were down-regulated. A total of 65 differential genes were screened out by the intersection analysis of 252 differential genes in 45 and 31 targets, and when these 65 targets were enriched, the results showed that FA and VA both regulated cell proliferation, redox process and apoptosis process by binding organelle enzyme binding, receptor activator activity, protein kinase binding, ATP binding and other synergistic biological functions by regulating MAPK signaling pathway, calcium signaling pathway, myocardial contraction, etc. DEGs is illustrated in Figure 2A–2C. The results also indicated that EGFR, PTPN1, ESR2, GSK3B, BACE1, PYGL, PTGS2 and APP were specifically expressed in skin aging, which was consistent with the above results of network pharmacology analysis.

### Synergic targets acting on skin aging of VA and FA

**Figure 3:**
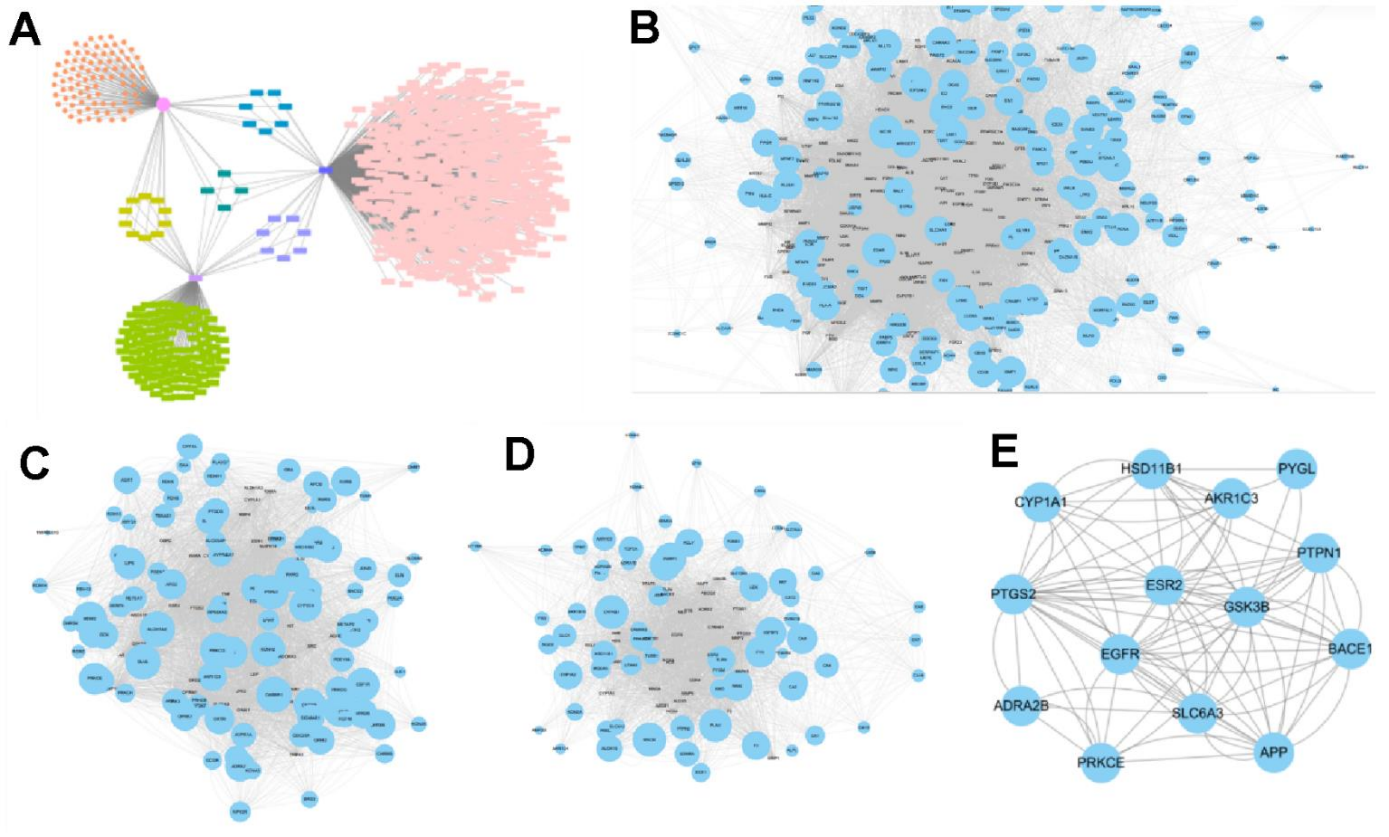
**Corresponding feature network diagram of targets of retinol, ferulic acid and skin aging.** (**A**) Distribution characteristic of targets of VA, FA and skin aging; (pink circle: targets of VA; pink rectangle: targets of skin aging; green rectangle: targets of FA). (**B**) PPI network of skin aging targets; (**C**) PPI network of VA targets; (**D**) PPI network of FA targets; (**E**) PPI network of Venn analysis of targets between VA, FA and skin aging.

Figure 3A showed the distribution characteristic of targets of retinol, ferulic acid and skin aging. As Figure 3B–3D, after PPI networks were introduced into Cytoscape software, a total of 25 synergic targets acting on skin aging of VA and FA were selected as key targets with node degrees were two-fold greater than the average node degree in the merge network, which consisted of 232 nodes and 680 edges. They mainly exerted photoprotection by targeting EGFR, PTPN1, ESR2, GSK3B, BACE1, PYGL, PTGS2 and APP (Figure 3E).

### Both VA and FA had higher docking scores with screened key targets

According to PPI analysis, EGFR, PTPN1, ESR2, GSK3B, BACE1, PYGL, PTGS2 and APP were the synergic key targets of VA and FA, and they were docked with VA and FA respectively to clarify the interaction relationships among the key targets. Van der Waals, conventional hydrogen bonds, carbon hydrogen bonds, pi-sigma, pi-sulfur, amide-pi stacked, and pi-alkyl were the principal intermolecular forces. As shown by the docking score, the results showed that VA and FA both had different degrees of docking with these key targets (Table 1), indicating VA and FA may have anti-skin aging effect by targeting those proteins. Result showed the 2D and 3D docking structure of VA and FA docked with top 5 synergic targets (AK1C3, GSK3B, ESR2, PTGS2, PTPN1). In current studies, VA exerted pharmacological effects greatly by Retinoic acid receptor/retinoid X receptor (RAR/RXR) signaling pathway, while activation of RAR/RXR pathway increased EGFR expression significantly and reduced transcription of PTGS2 (protein COX-2) [25, 26]. FGFR1 was well known core targets of FA, inhibition of COX-2 prior to activation of FGFR1 in the transgenic mice also resulted in decreased of FGFR1 amplified-induced initiation of hyperplastic lesions [27, 28]. Proliferation of FGFR1 and PTGS2 played a role in promoting the course of the disease.

**Table 1:** Docking score of VA and FA with screened key targets.

**Target**	**Compound**		**Target**	**Compound**	
	**Retinol**	**Ferulic_acid**		**Retinol**	**Ferulic_acid**
AK1C3	-9.5	-7.2	PKCE	-9	-6.9
BACE1	-7.7	-6.4	PTPN1	-7.2	-7
APP	-7	-6.3	PYGL	-7.7	-6.6
EGFR	7.7	-6.2	SLC6A3	-6.8	-6
GSK3B	-8	-6.8	ESR2	-8.4	-6.5
HSD11B1L	-6.4	-5.8	PTGS2	-7.9	-7.4

### Effects of retinol on HaCaT cell proliferation with ferulic acid

**Figure 4:**
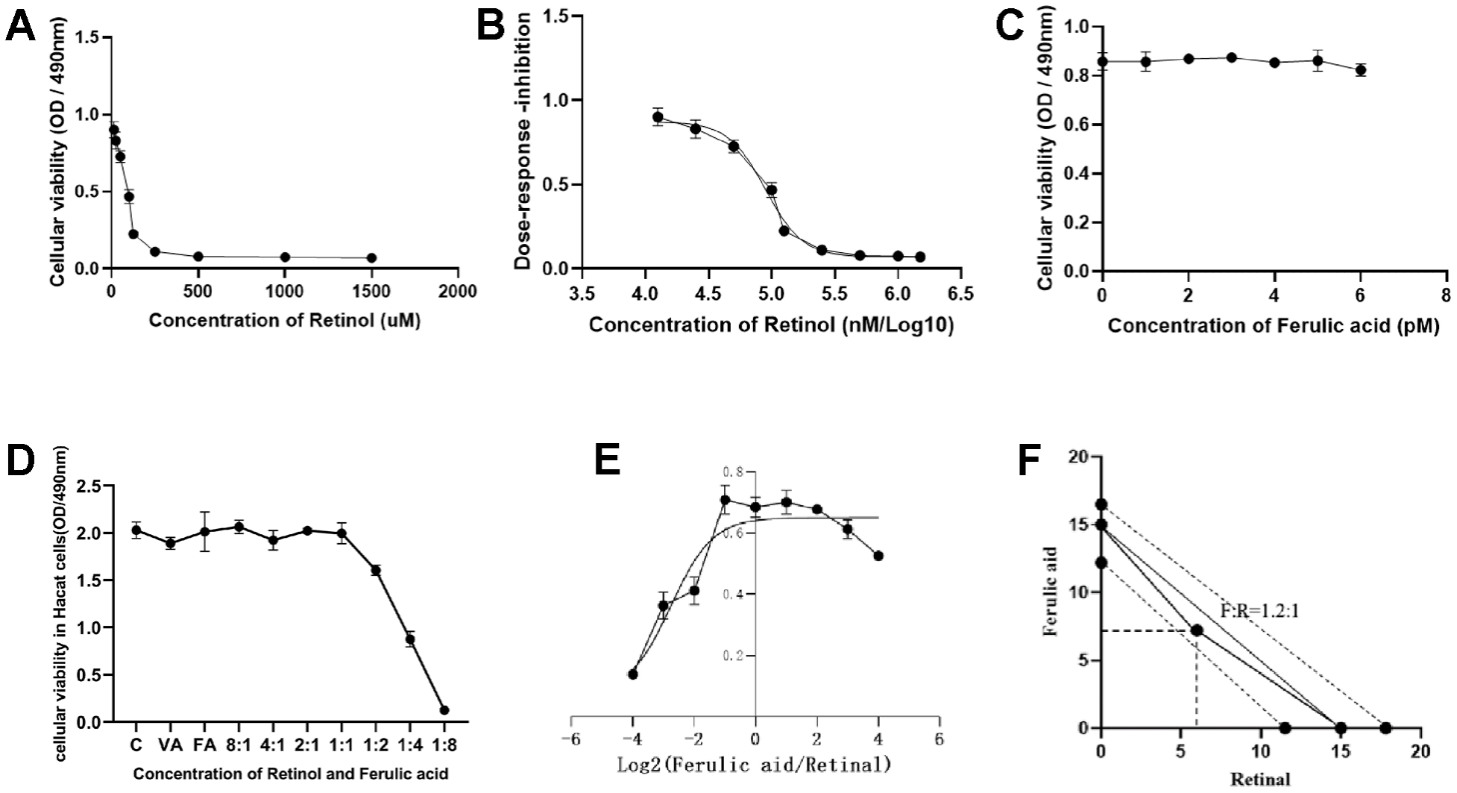
**The effects of varying doses of VA and FA on the proliferative activity of HaCaT cells and UVB-induced aging in HaCaT cells.** (**A**) Cellular viability of VA; (**B**) Dose-response inhibition of VA; (**C**) Cellular viability of FA; (**D**) Cellular viability of FA and VA in HaCaT cells; (**E**) Effects of different ratios of VA and FA on UVB-induced aging cells proliferation (X-axis presented concentration of VA and FA, Y-axis presented viability of cells); (**F**) Isoradiometric analysis of VA and FA for UVB-induced aging cells proliferation (X-axis presented concentration of VA, Y-axis presented concentration of FA).

As shown in Figure 4A, the viability of HaCaT cells gradually decreases as the concentration of VA increases. When the concentration of retinol reached 1500 μM, HaCaT cell viability drops to a minimum. According to Figure 4B, the IC_50_ concentration of VA is 90nM. That is, when the concentration of retinol reaches 90nM, it can cause HaCaT cells in the death of 50%. As shown in Figure 4C, different concentrations of FA had no effect on the proliferative viability of HaCaT cells.

According to the MTT method, administration with mixed VA and FA in the ratio of 1:0, 1:1, 1:2, 1:4, 1:8, 0:1 showed no significant difference in survival compared with normal HaCaT cells (Figure 4D). As shown in Figure 4E, based on the isoradiation principle, mixed VA and FA in the ratio of 1:0, 8:1, 4:1, 2:1, 1:1, 1:2, 1:4, 1:8, 0:1. Combining with FA, the anti-aging efficacy of retinol was enhanced while cell irritant is attenuated at an optimal ratio. The results showed that the different doses of FA promoted the cytoprotective effect of retinol. Isoradiometric analysis showed that when FA:VA=1.2:1 (120 nM:100 μM) (Figure 4F), the two drugs had the effect of synergistic protection on HaCaT cells. Therefore, adopted the ratio of FA:VA=1.2:1 for subsequently experimental verification. The dose-response curve shows a combination of VA and FA, the reaction index of which is 0, manifests itself as a synergistic effect, therefore, ferulic acid and retinol have a synergistic effect.

### Response surface analysis of ferulic acid to retinol verified the optimal ratio

**Figure 5:**
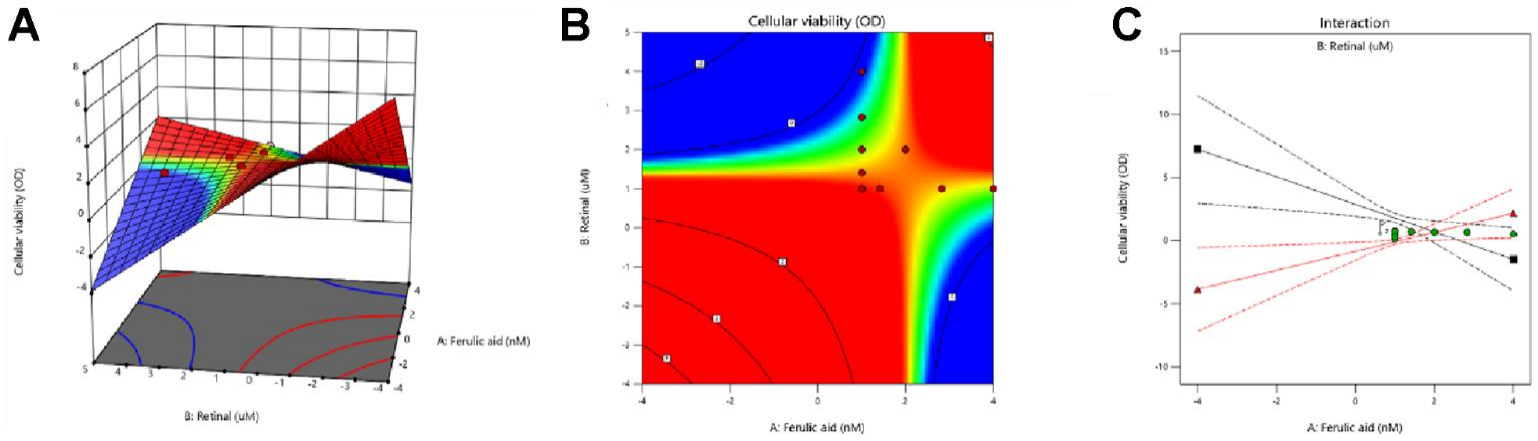
**Response surface analysis was used to verify the optimal ratio of FA to VA.** (**A**) 3D response surface model diagram of administrated groups; (**B**, **C**) Response surface plane projection of VA and FA.

As shown in Figure 5A–5C, the dose-response curve was described by quadratic polynomial which showed the combination of VA and FA. The response index indicated the intensity of their interaction, to illustrate the degree of interaction among the doses of drug administration. The result was presented by subtracting the response index by 1 to 0 as a typical additive. In the 3D response surface model diagram, the color shade indicated the intensity of the interaction between the two drugs. When the response index was close to 0, the drug was shown as an additive effect; when the response index was greater than 0, it was manifested as antagonism; When less than 0, it manifested as synergy. The higher the index value, the greater the intensity of the interaction was, and the response surface plot appeared to be colored close to either light or dark. The X-axis represented different doses of VA, the Y axis represented different doses of ferulic acid, and the Z axis represented a gradient change of the pairs of drugs in the case of fixed IC_50_ of one drug. The inhibition rate of HaCaT cell activity (IC_50_) was used as the evaluation index, and the three-dimensional response surface model was fitted. The synergistic effect was significant when the ratio of ferulic acid and retinol was between 0.671:1~1.657:1 (nM: μM).

### β-galactosidase staining monitored the senescent phenotype on HaCaT cells with FA and VA

**Figure 6:**
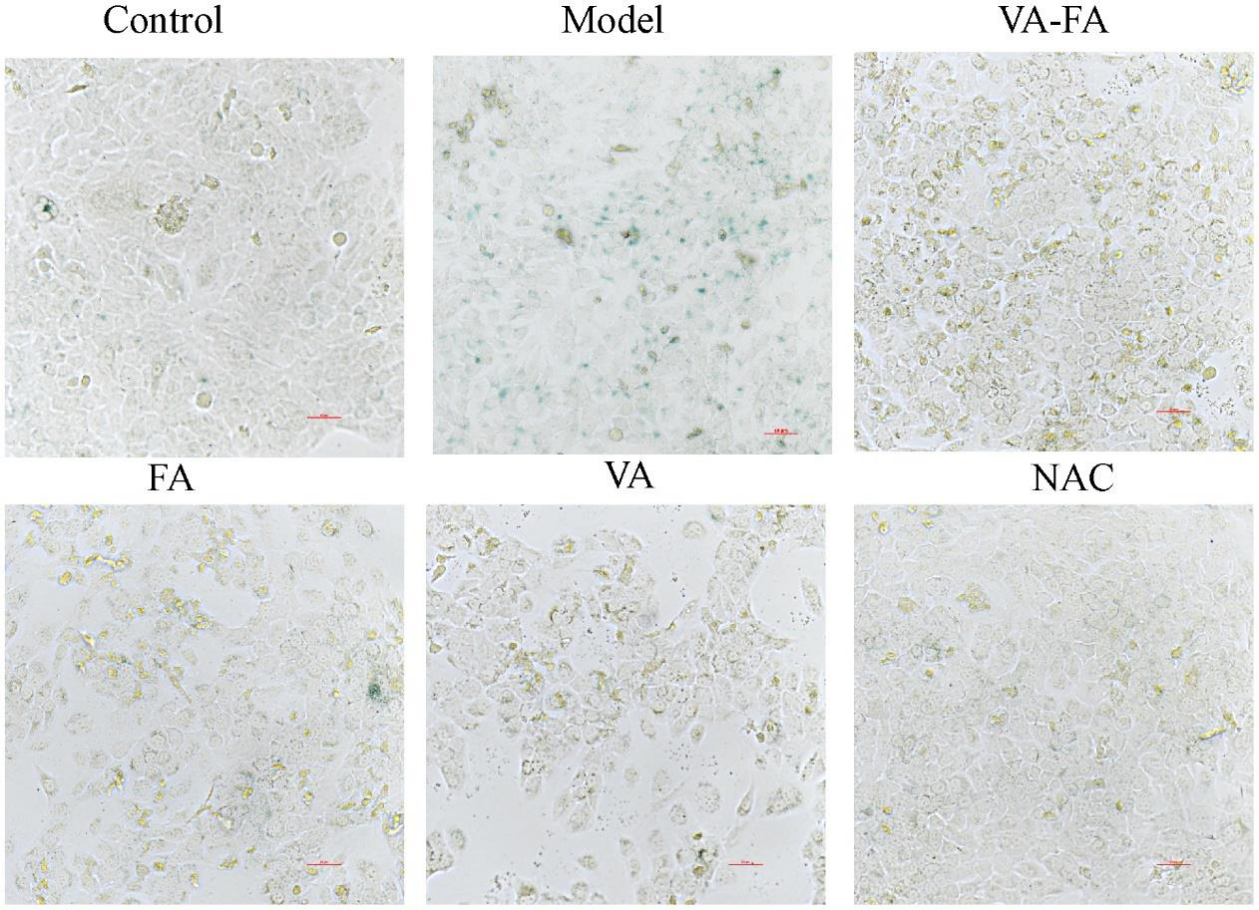
**Senescence-associated-β-galactosidase (SA-β-gal) staining.** Control: normal group; Model: UVB radiation group; VA-FA: mixture of VA (120 μM) and FA (100 nM); VA: only retinol (120 μM); FA: only ferulic acid (100 nM).

To identify senescent cells, SA-β-gal staining is a commonly used method [29]. The higher the degree of senescence, the more blue-colored cells appear in the visual field. As shown in Figure 6, there were significantly more senescent (SA-β-gal positive) HaCaT cells after UVB irradiation (model group), while cell morphology contracted and the intercellular gap widened. Following VA-FA and NAC administration, there were significantly fewer senescent cells in the visual field, and the intercellular space and cell shape were comparable to those of the normal group (control group). Only the administration of VA and FA produced reduced cells and greater intercellular spaces, but fewer stained cells overall.

### Effects of retinol and ferulic acid on reactive oxygen species in cells

**Figure 7:**
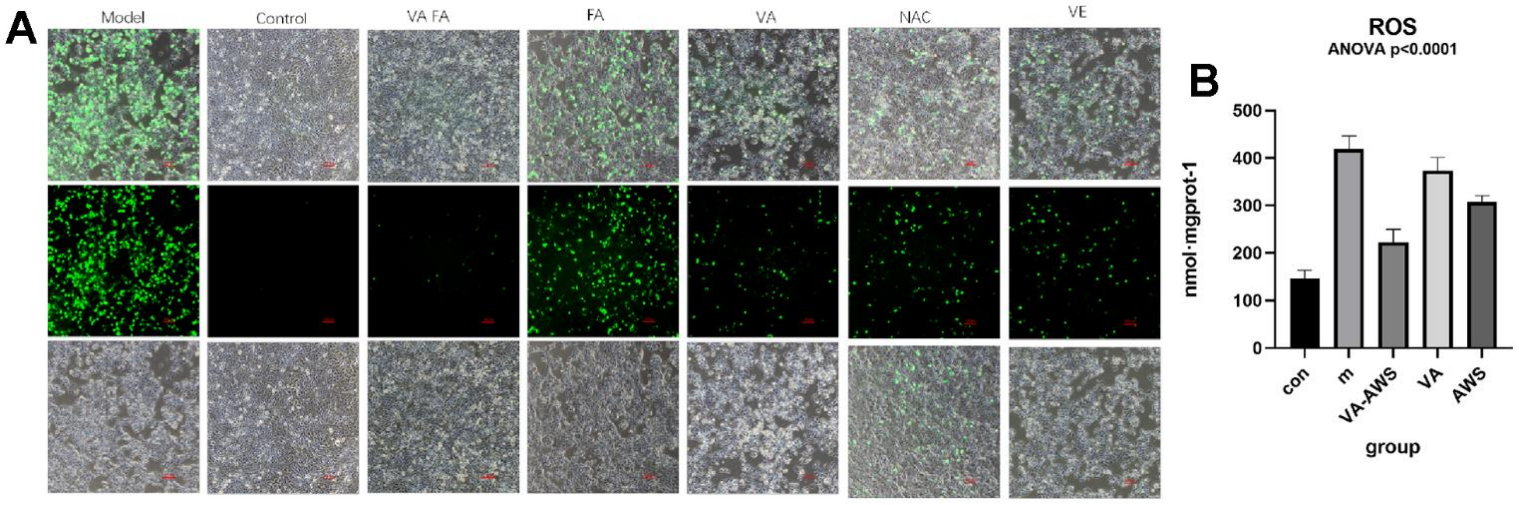
**Effect of administration groups on reactive oxygen species.** (**A**) Representative fluorescence images of HaCaT cells with different compounds addition; (**B**) Fluorescence intensity of ROS level among FA and VA addition groups of HaCaT cell (Control: normal group; Model: UVB radiation group; VA-FA: mixture of VA (120 μM) and FA (100 nM); VA: only retionol (120 μM); FA: only ferulic acid (100nM)).

Photoaging HaCaT cell was induced by UVB, which was loaded with fluorescent probes of the reactive oxygen species (ROS) detection kit. The images of each group of cells were observed at FITC mode (Figure 7). Examined under a microscope, the cell morphology of the UVB irradiation group had obvious changes. The cell boundaries after irradiation were not clear, the gap increases, shrinks, rounds, and even cell ruptures. The fluorescence was strong, indicating that the ROS level was high. Administrated with VA, FA, N-acetylcysteine (NAC), and vitamin E (VE) separately, the damaged cells recovered and fluorescence decreased to some extent. The cell contours of the retinol and ferulic acid groups were clearer than those in the model group, the number of normal cells was significantly increased, and the fluorescence was significantly reduced. Numerically, there were significant differences between the ROS levels of cells in the model group and the normal group, the co-administration group (p-value<0.0001), and with the ferulic acid group (p-value<0.01). It was indicated that the combined administration of retinol and ferulic acid had a significant inhibitory effect on UVB-induced photoaging of HaCaT cells. VA and FA slowed down UVB-induced HaCaT cell photoaging by reducing ROS.

### Detection of cellular SOD, MDA, GSH, CAT

The levels of MDA, SOD, GSH, CAT (Table 2) reflected the oxidative stress response of HaCaT cells after administration. What could be recognized is that compared with the FA group, normal group and model group, there were significant differences in all indexes (p<0.0001). Indicating that after synergistic drug delivery, the level of MDA caused by oxidative stress was significantly reduced, and the content of SOD, GSH and CAT was significantly increased. After the administration of VA and FA, respectively, it also had a certain effect of increasing the GSH content in photoaging model cells. FA also had the effect in increasing CAT and reducing MDA levels. It suggested that FA in synergizing with VA could enhance the protective effect on photoaging keratinocytes through antioxidant. All experiments followed the principle of complete randomization. Comparisons between groups were analyzed using ANOVA, all results were expressed as mean ± standard deviation, and the above calculations were performed using GraphPad Prism software.

**Table 2:** Effects of retinol and ferulic acid on SOD, CAT, GSH, MDA, ROS in UVB-induced photoaging HaCaT cells $(\bar{x}\pm s,\;n=3)$.

**group**	**SOD (U·mgprot^-1^)**	**GSH (U·mgprot^-1^)**	**MDA (U·mgprot^-1^)**	**CAT (nmol·mgprot^-1^)**
Control	1.881±0.07079***	45.52±2.225***	15.24±2.873***	289.1±38.49***
Model	0.4823±0.02325	25.16±2.227	40.07±5.796	66.5±8.996
VA-FA	1.473±0.2013***	39.34±1.245***	17.95±4.329***	303.1±19.15***
VA	0.6247±0.1191	29.75±3.268**	31.2±3.916	140±10.92
FA	0.7303±0.1197	30.72±1.168**	28.26±3.496*	186.7±11.82*

Note: Compared with the photoaging model group, ***P<0.0001, **p<0.01, *P<0.05.

### A higher correlation between FA and VA groups and normal groups in HaCaT cells

**Figure 8:**
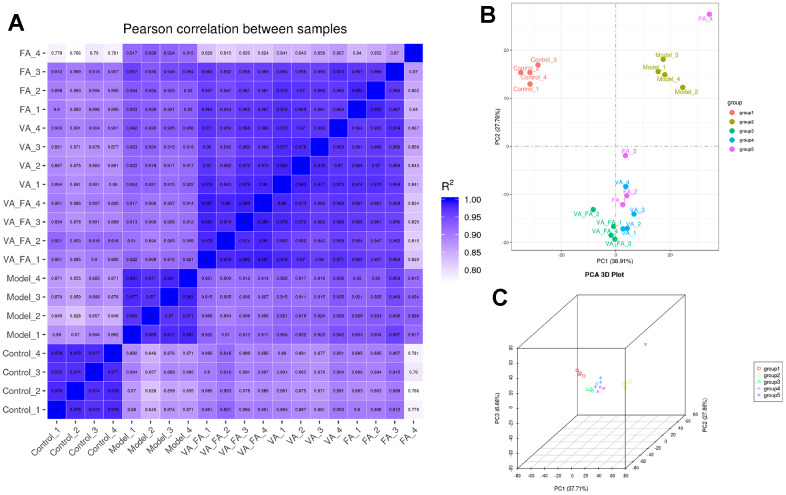
**Significant intergroup differences among the cells in each group.** (Group 1: control group; group 2: UVB induce aging group; group 3: VA-FA addition group; group 4: VA addition group; group 5: FA addition group). (**A**) Pearson correlation analysis in HaCaT cells; (**B**) Two-dimensional principal component analysis in HaCaT cells; (**C**) Three-dimensional principal component analysis in HaCaT cells.

Through Pearson correlation analysis, Figure 8A, the gene of HaCaT cells in each of group shows high similarity coefficient, and there were significant differences between groups. Through principal component analysis, Figure 8B, 8C showed a trend of aggregation in the normal group, model group, VA and FA administration group, and the relative dispersion between groups. The relative position of VA and FA administration group was between the normal group and the model group, and was closer to the normal group than that of the drug group alone. It was shown that the abnormal expression of photoaged HaCaT cells induced by the most UVB induced by VA and FA administration had a significant callback effect.

### Effects of retinol synergistic ferulic acid on gene expression in HaCaT cells

**Figure 9:**
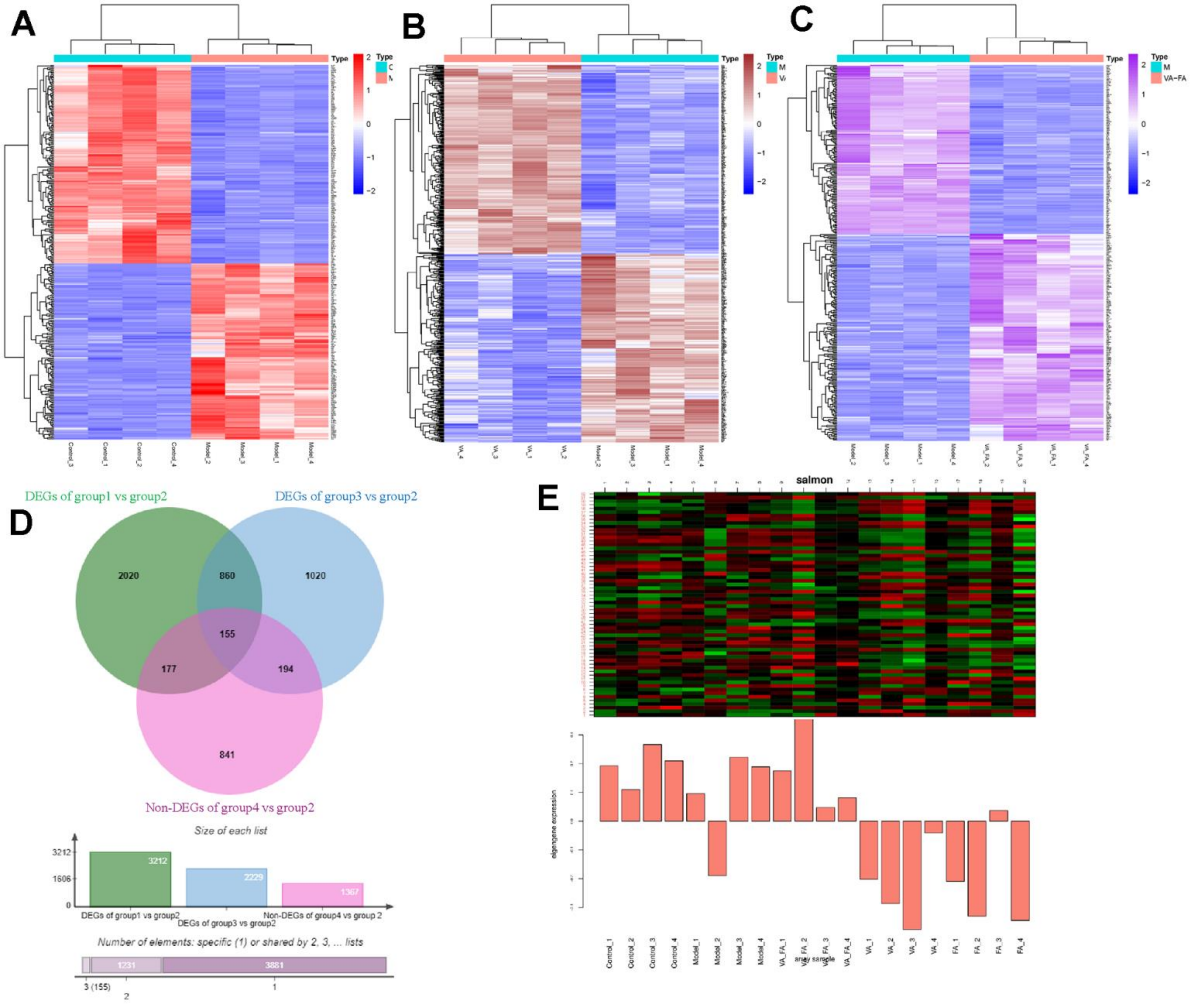
**Significant differences in gene expression between the HaCaT cells from different groups and the model group.** (Group 1: control group; group 2: UVB induce aging group; group 3: VA-FA addition group; group 4: VA addition group; group 5: FA addition group). (**A**–**C**) Heat map of DEGs in groups of control vs model, VA vs model, VA-FA vs model; (**D**) Venn plot of DEGs in HaCaT cell among groups; (**E**) Heat map of DEGs in HaCaT cell among groups.

The heat map of each group was visible (Figure 9A–9C and Supplementary Table 6). Compared with the UVB radiation HaCaT cell model group, the gene expression difference in the cells of the FA cooperating with VA administration group was revealed. It was closer to that of the normal group cells (Figure 9E). Venn plots of the DEGs (Supplementary Table 7) sets obtained by the two groups show the genes that were common and unique between each gene set (Figure 9D). A total of 332 genes had significant gene expression changes between the normal group and the model group. 349 genes had significant gene expression differences between the VA-FA administration group and the model group. However, 155 genes in the co-administration group, normal group and model group had distinct differences in gene expression (Supplementary Table 8). These genes were different from the effects caused by VA administration and the key genes involved in the reduction of the keratinocytes photoaging degree by FA in combination with VA. PTGS2 was identified as core target by comprehensive analysis of network pharmacology, comparing VA-FA groups and Model groups, log (FC)=-3.22. At the same time, it was the gene with significant expression differences in RNA sequencing. The results showed that the VA-FA group vs model group had more genes in common with the genome gene set than the normal group VS model group. The VA-FA group gene set was closer to the normal group.

### Regulation of COX-2 by ferulic acid in concert with retinol in HaCaT cells

In our present investigation, we explored into how VA and FA affected COX-2. UVB radiation significantly elevated COX-2 expression in HaCaT cells, as Table 3 illustrated. Following UVB radiation, the expression of COX-2 was down-regulated in the group treated with VA and FA alone, whereas it was down-regulated in the group treated with VA and FA combined.

**Table 3:** Effects of retinol and ferulic acid on expression of COX-2 in UVB-induced photoaging HaCaT cells $(\bar{x}\pm s,\;n=3)$.

**group**	**COX-2 concentration (OD)**	**Number of values**	**F**	**P-value**
C	0.5928±0.0057***	3	170.9	<0.0001
M	0.8043±0.0405	3		
MIX	0.4101±0.0056***	3		
FA	0.5036±0.0062***	3		
VA	0.6167±0.0024	3		
NAC	0.5317±0.0109**	3		

Note: Compared with the photoaging model group, ***P<0.0001, **p<0.01, *P<0.05.

### Regulatory gene function of VA and FA by enrichment analysis

**Figure 10:**
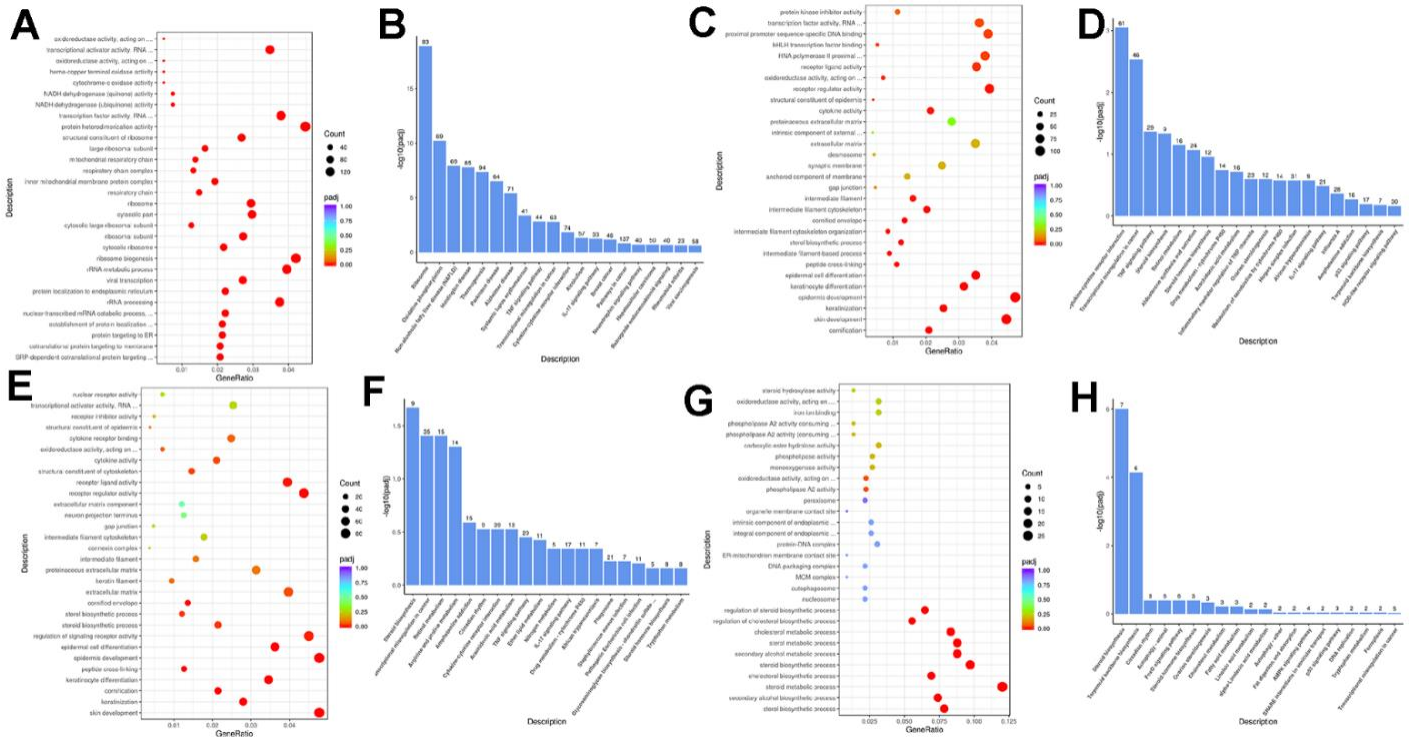
**The combined action of retinol and ferulic acid regulates biological processes that contribute to the protection of photoaging cells.** (**A**, **B**) Bubble diagram and bar chart of DEGs in groups of control vs model; (**C**, **D**) Bubble diagram and bar chart of DEGs in groups of VA-FA vs model; (**E**, **F**) Bubble diagram and bar chart of DEGs in groups of VA vs model; (**G**, **H**) Bubble diagram and bar chart of DEGs in groups of FA vs model.

Enrichment analysis was performed by comparing the genes of each group with the photoaging HaCaT cell group. As shown in Figure 10A–10H and Supplementary Table 9, the discrepancy between the normal group and the model group was mainly reflected in oxidative phosphorylation, oxidoreductase activity, transcription-related pathways, etc. The synergistic drug delivery group acted on biological processes such as oxidoreductase activity, skin growth, keratinization, and steroid biosynthesis. It suggested that the commonality between the groups was reflected in the regulation of redox reaction, skin growth and keratinization, thereby producing synergistic protection.

## DISCUSSION

In majority of researches, aging skin exhibits morphological changes, particularly in the dermis and epidermis. Deterioration of the skin barrier, wrinkles, pigmentation, chronic inflammation, and infection risk are all associated with aging skin [30]. Important contributors to skin aging were endogenous metabolism or UVR that produced free radicals and reactive oxygen species (ROS) [31]. The accumulation of ROS induces oxidative stress, mitochondrial DNA damage [32], guanine oxidizing, protein carbonylation [33], inflammatory responses and cells damaging [4]. The accumulation of ROS induces level of proinflammatory cytokines, IL-6, COX-2, TNF-α, etc. [4, 34]. ROS induced by ultraviolet irradiation caused JNK and p38 to activate transcription of AP-1 in keratinocytes and fibroblasts. AP-1 binds to the PTGS2 gene promoter, increasing the transcription of COX-2 [8]. Gene PTGS2 encodes the enzyme cyclooxygenase-2 (COX-2), which plays a significant role in orchestrating chronic inflammation, cellular proliferation, apoptosis, and immunity [35]. These factors make the skin barrier state unstable and accelerate skin aging. The skin is susceptible to various redox reactions [36]. Compensatory defensive enzymes and non-enzymatic antioxidants are brought about by the increase in ROS concentration of epidermal cells, which can be used as an important direction for the study of anti-skin aging [37]. Therefore, oxidative stress plays a pivotal role in cellular senescence, inflammation, and cell dysfunction.

VA is a powerful free radical scavenger observed in high concentrations of the epidermis and dermis, protecting skin against ROS-induced oxidative damage [13]. However, UV-induced damage may reduce the antioxidant defense system in skin. Some studies have proposed that topical application of VA to skin may reconstitute a cutaneous antioxidant system in inhibiting UV-induced oxidative damage [10]. Nevertheless, the antioxidant effect of VA is strictly associated with the characteristics of the formulation, mainly the concentration. Accordingly, stable aqueous formulations containing 15%-20% VA, 1% vitamin E, and 0.5% FA at a pH less than 3.5 have been developed [38]. These formulations may protect skin against UV-induced damage because they are more available that promote absorption and delivery of VA to skin. However, neither study evaluated whether these formulations induce histological and biochemical alterations in human skin *ex vivo*.

In this study, the degree of medication efficacy and interaction between VA and FA on aging skin was predicted by network pharmacology. PPI network analysis represented that VA and FA were acted on similar target proteins of skin aging. GSEA analysis showed synergic biological function of VA and FA in regulating MAPK signaling pathway, calcium signaling pathway, cardiac muscle contraction acting on EGFR, PTPN1, ESR2, GSK3B, BACE1, PYGL, PTGS2 and APP. The heat map and volcano plot showed significant discrepancy of DEGs in groups of normal and UVB irradiation groups. The high docking score between VA, FA and 5 top synergic targets (AK1C3, GSK3B, ESR2, PTGS2, PTPN1) verified the methodology.

The cell experience was carried out to inquire into the composition and dose applying VA and FA in skin photoaging. Response surface analysis and the MTT test showed that while VA did not considerably enhance photoaging HaCaT cell morphology, it did partially improve it when given alone. The maximum value of recovery for UVB-induced photoaging cell viability was attained when the ratio of FA to VA reached 0.671:1~1.657:1 (μM:nM), as indicated by the inhibition rate of HaCaT cell activity (IC_50_). The synergistic group’s cells showed a dramatic drop in fluorescence under a microscope in ROS detection, and the cell morphology recovered significantly. The recovery state of VA-FA group cells was better than that of the positive drug groups, NAC and VE groups. The VA group cells exhibited lower fluorescence intensity and less noticeable cellular morphology recovery. Based on the indicators of oxidative stress such as MDA, SOD, GSH, and CAT, the levels of VA-FA group cells in MDA, SOD, CAT were upregulated. The outcome showed the restoration of antioxidant defense mechanisms in addition to the scavenging of lipid peroxides and free radicals. The fall in GSH levels suggested that the structure and function of cell membranes were less damaged by oxidative interference. Each treatment group’s pharmacological outcomes matched the medication efficacy routes that network pharmacology anticipated. Therefore, it appears that the combination of VA and FA increased the activity of antioxidant enzymes, which helped to remove the oxidative stress brought on by photoaging. This achievement of oxidative-reductive balance helped maintain cellular and organelle homeostasis, thus preventing photoaging of skin cells.

Principal component analysis (PCA) along with heatmap and volcano plot visualization in RNA transcriptome was conducted to illustrate the expression differences of genes among different cell groups. By comparing the overall gene expression patterns of HaCaT cells in various groups, it was observed that there was a high similarity within each group, whereas significant differences exist between the groups. A total of 155 genes exhibited significant differences in gene expression among the co-administration group, normal group, and model group cells. Notably, the gene PTGS2 demonstrated a high binding affinity with VA and FA in molecular docking analysis. It was believed that PTGS2 played a crucial role in the collaborative effect of retinol and ferulic acid regulating the process of UVB-induced photoaging in HaCaT cells. The expression of PTGS2 associated with skin aging and melanoma, which in the co-administration group was reduced and overexpressed in UVB-induced photoaging cells. The inhibition of PTGS2 could suppress the cell proliferation and migration in malignant melanoma by overexpression of eukaryotic translation initiation factor 3 subunit B [39]. The DEGs in each group of cells were enriched in processes involving the regulation of oxidation-reduction reactions, skin growth, and keratinization. It is evident that the regulation of oxidative stress responses constitutes an essential mechanism through which the combined administration of retinol and ferulic acid effectively mitigates skin aging.

In summary, for the first time, we have discovered co-administration with ferulic acid at specific proportions, the utilization efficiency of retinol can be significantly enhanced, overcoming its limitations. Through cellular experiments, it was observed that co-administration primarily achieves the maintenance of cellular redox homeostasis by scavenging free radicals and enhancing antioxidant defense capabilities. The discovery of the PTGS2 gene’s involvement in oxidative-reduction and inflammatory responses during HaCaT cell photoaging process suggests that PTGS2 is a key regulatory gene. PTGS2 is the core target for treating oxidative stress in skin photoaging, holding promise for therapeutic interventions.

## CONCLUSIONS

In this study, the regulatory pathway and mechanism of FA in synergizing with VA were predicted by network pharmacology. Through cell experiments, the pharmacodynamic effect and optimal ratio of VA and FA on UVB-induced photoaging of HaCaT cells were explored. FA blunted to the stratum corneum stimuli from VA, while two drugs synergistically enhanced photoaging-resistance capacity. At the concentration of VA:FA of 100 μM:120 nM, the content of MDA and ROS decreased significantly, and other indicators reflecting the level of oxidative stress such as SOD, GSH, and CAT also increased to a level close to normal cells. Moreover, the morphology of photoaged cells recovered after synergistic administration. RNA sequencing results exhibited that the transcription level of cells in the co-administration group was closer to the normal group. A total of 155 the DEGs specifically synergistically acted on photoaged HaCaT cells. Noteworthy, PTGS2 showed high affinity with VA and FA in molecular docking, and was verified by transcriptomics as a key expression gene. The mechanism of VA and FA in alleviating the photoaging process is mainly involved in restoring anti-oxidative stress system and anti-inflammatory.

## MATERIALS AND METHODS

### Collection of known and predicted targets of VA and FA

Known targets of VA and FA were collected from The Traditional Chinese Medicine Systems Pharmacology Database (TCMSP, http://lsp.nwu.edu.cn/) and the Drugbank Database (https://go.drugbank.com/). Predicted targets of both components were collected from SwissTargetPrediction database (http://swisstargetprediction.ch/). In addition, targets identified in published literature were also collected.

### Collection of human skin aging disease targets

The targets of skin aging were obtained from the GWAS database (https://www.ebi.ac.uk/gwas/) and GeneCards Database (https://www.genecards.org/) according to the MESH of diseases including “Skin Aging”, “Aging, Skin”, “Solar Aging of Skin”, “Photoaging of Skin”, “Skin Wrinkling”, “Skin Wrinklings”, and “Wrinkling, Skin”. The disease targets related to human skin aging were filtered based on a correlation score in databases greater than 0.5 or 80 points.

### Venn analysis

Intersection analysis was performed separately on the target proteins of VA and FA associated with skin aging targets. The intersecting targets were considered as the common targets for the effects of VA and FA on skin aging-related diseases. Subsequently, we performed biological functional analysis on the two intersecting targets separately using the DAVID database. We screened for targets directly connected to mechanisms of skin aging diseases, such as regulating inflammation processes, antioxidant functions, and immune regulation. These targets were considered as the common targets for both VA and FA in skin aging. These biological processes and targets form the foundation for the synergistic action of VA and FA.

### Gene set enrichment analysis (GSEA)

Using R 4.2.0 software, Gene Set Enrichment Analysis (GSEA), a knowledge-based method for analyzing genome-wide expression patterns, was carried out on hub targets. It included hub target pathway enrichment from the Kyoto Encyclopedia of Genes, Genomes (KEGG) and Gene Ontology Annotation (GO). Enrichment bubbles were also constructed through R software 4.2.0 with top 20 items with significant differences (adjusted P<0.05).

### Identification of differentially expressed genes (DEGs) in skin aging

Expression profiling by high throughput sequencing with series number GSE182673 based on platform GPL16791 (Illumina HiSeq 2500, Homo sapiens) was downloaded from the GEO database. This dataset contained 50 skin aging samples and 25 normal samples, which identified 881 DEGs. DEGs between skin aging groups and normal group were respectively analyzed using the limma package in R. Fold changes (FCs) in the expression of individual genes were calculated. DEGs with adj Pval < 0.05, log FC> 1 for up regulated genes and log FA< − 1 for down regulated genes were considered as significant. Subsequently, Venn analysis was performed on the DEGs and selected potential therapeutic genes. The resulting common genes were considered as the synergistic targets of VA and FA. The DAVID database was then used to carry out a second round of biological functional enrichment analysis and KEGG pathway enrichment analysis, then investigated the pharmacological effects and molecular mechanisms of the synergistic action of VA and FA.

### Protein–protein interaction analysis and molecular docking

According to the correlation score>0.4, the Protein–Protein Interaction (PPI) network was built by targets of VA, FA and skin aging, which were obtained from Search Tool for the Retrieval of Interacting Genes/Proteins (STRING, a free biological database) (https://cn.string-db.org/cgi/) respectively. PPI network nodes were introduced into Cytoscape 4.0 software for visualization. The main targets with top 10 values were screened after network topology analysis. Then VA and FA were molecularly docked with the top 5 key targets through Discovery studio software.

### Weighted gene co-expression network analysis

Using WGCNAR package, GEO expression file was applied for weighted gene co-expression network analysis (WGCNA). The association between the clinical characteristics and expression modules was investigated using WGCNA. Module eigengenes (MEs) were defined as the first principal component of each gene module and adopted as the representative of all genes in each module. Gene significance (GS), as the mediator p-value (GS=lg P) for each gene, represented the degree of linear correlation between gene expression of the module and clinical features. Survival-related modules were defined according to P≤0.01 and the higher GS value was extracted for further analysis.

### Molecular docking on hub targets of VA and FA

The protein structures of hub targets were downloaded from the PDB Database (https://www.rcsb.org/). The structural files of the key active compounds of VA and FA were downloaded from the PubChem Database (https://pubchem.ncbi.nlm.nih.gov/) and were saved in *SDF format. The abovementioned structures were added hydrogens, and the charges were firstly calculated by Discovery studio software (2.5 version), and then they were docked through the Discovery studio software, the model with the highest binding score value was selected, and their binding structure finally was visualized by Discovery studio software.

### Reagents and materials

VA was bought from Shanghai McLean Biochemical Technology Co., Ltd. (Shanghai, China), and FA was from Shanghai Yuanye Technology Co., Ltd. (Shanghai, China).

Human immortal keratinocyte cell line (HaCaT) was purchased from American Type Culture Collection (Rockville, MD, USA). The 10 cm^2^ cell culture dishes, 6-well plates, and 96-well plates were purchased from Corning Incorporated (Corning, NY, USA). DMEM Medium, fetal bovine serum, 100 U/ml penicillin, and 100 μg/ml streptomycin were purchased from Gibco (Thermo Fisher Scientific Co., Ltd, Waltham, MA, USA; Grand Island, NY, USA). Reactive oxygen species (ROS) assay kit, GSH, SOD, MDA and assay kit were purchased from Beyotime (Beyotime Biotechnology Co. Ltd., Shanghai, China). Catalase Activity Assay Kit was purchased from Abcam (Abcam (Shanghai) Trading Co., Ltd., China; ab83464). 3-(4,5-dimethylthiazol-2-yl)-2,5-diphenyltetrazolium bromide (MTT) was purchased from Sigma-Aldrich (MTT, Sigma-Aldrich, St. Louis, MO, USA). Senescence-associatedβ-galactosidase (SA-β-gal) Stain Kit was produced from Solarbio Technology Co. Ltd. (Beijing, China). Human Cyclooxygenase-2 (COX-2) ELISA Kit was produced from Cusabio Technology, LLC (Wuhan, China). RNA Nano 6000 Assay Kit of the Bioanalyzer 2100 system (Agilent Technologies, Santa Clara, CA, USA) was used.

### Cell culture

Human immortalized epidermal cells (HaCaT) were cultured in DMEM (high glucose) medium with 10% fetal bovine serum and 1% penicillin-streptomycin double antibody. They were maintained in a cell incubator with 5% CO_2_ at 37° C. Subculture was performed when the cell confluency reaches 80%.

### UVB-irradiation model and validation

HaCaT cells were cultivated to 80%–90% confluence, which were irradiated at 302 nm and subjected to UVB radiation at varying intensities (18-54 mJ/cm^2^) following a PBS wash. After radiation, the cells were cultured in full medium supplemented with 10% serum.

### Cytotoxicity assay

Cytotoxicity was detected by MTT assay. In brief, HaCaT cells were seeded into 96-well plates with a density of 1×10^4^/well. After treatment, 10 μL of 5 mg/mL MTT solution was added to each well and then cells were incubated in the dark for 4 h. After removing the cultured medium, the crystals were dissolved in 150 μL of DMSO. The absorbance was measured at 490 nm with a BioTek plate reader (American Boten Instrument Co., Ltd., Winooski, VT, USA). Using ferulic acid and retinol cells, respectively, the toxicity of both components to HaCaT cells and IC_50_ values was explored.

### Effects of VA and FA on HaCaT cell proliferation

HaCaT cells (1×10^5^ cells/well) were seeded in 96-well transparent plates. After cell adhesion, the original medium was aspirated. 100 μL complete medium was added to the blank group. The gradient concentration groups complete medium with 4μM~1.5mM and complete medium with 1pM~1mM ferulic acid were separately added to cells. After 24 h of incubation, 5 mg/mL Methyl Thiazolyl Tetrazolium reagent was added to each well, the supernatant was aspirated after 4 hours, and dimethyl sulfoxide (DMSO) was added to each well to detect absorbance. Therefore, the influence of VA and FA on the proliferation of HaCaT cells was investigated, and calculated the IC_50_ dose. The experiment was repeated 3 times. The experimental data were statistically analyzed using GraphPad Prism 9, using ordinary one-way ANOVA for multiple comparisons, and Tukey’s multiple comparisons teat, p<0.05.

### Effects of VA and FA on HaCaT cells

HaCaT cells (1×10^5^ cells/well) were seeded in 96-well plates, and after the cells were adhered, discarded the original medium. The blank group and the model group were added complete medium. In administration group, VA and FA were set according to the value of IC_50_, one of the drug doses was set to the concentration of IC_50_. Using radiation design, the two-dimensional coordinates were divided into 8 equal parts, and 9 different proportions (1:0, 8:1, 4:1, 2:1, 1:1, 1:2, 1:4, 1:8, 0:1) were obtained, carried out ratio test separately. Positive drug group were treated with N-acetyl cysteine (NAC) and Vitamin E (VE) in concentration of 2.5 mM and 1.56 μM. Incubated for 24 h, both the model group and the experimental group were exposed with UVB rays (54mJ/cm^2^). The activity of HaCaT cells in different groups was measured. Subsequently, the quadratic polynomial was used to describe the dose-response curve of the combination of FA and VA, and the response index indicated the interaction strength. Subtract the response index by 1 and take 0 as a typical addition effect for easy plotting. In the 3D response surface model diagram, the color shade indicated the intensity of the interaction between the two drugs. When the response index was close to 0, the drug was shown as an additive effect; when greater than 0, it was manifested as antagonism; when less than 0, it manifested as a synergistic effect. The higher the index value, the greater the intensity of the interaction, and the response surface plot appears to be colored close to either light or dark. Matlab software was used to fit the model and determine the parameters. Taking the cell viability rate as the evaluation index, the three-dimensional response surface model was fitted.

### Pharmacodynamic response surface analysis of the interaction between VA and FA

NLMAZ is an extension to the mixed amount with zero amount observation model that it allows for a different concentration-pharmacodynamic response in two or more drugs [40]. The absorbance value obtained from cell experiment were input to describe the dose-response curve by quadratic polynomial, following each treatment with two drugs in combination as well as in the case of untreated positive control and negative control [41]. This stepwise regression method was used to construct the index and evaluate the model of different proportions of drugs. Then introduced response index to indicate the interaction strength. The three-dimensional response surface plots were presented with Origin9.1 software (OriginLab Corporation, Northampton, MA, USA) and according to the statistical strategies, calculated the best ratio.

### Staining for β-galactosidase activity

HaCaT cells were plated into 6-well plates and cultured for 24 hours. Blank control, VA-FA, VA, FA, and NAC were introduced to the respective plates following UVB irradiation, and the plates were then incubated for 24 hours at 37° C. After 24 h, the cells were fixed and stained according to the β-galactosidase activity kit instructions, and they were then left overnight to incubate at 37° C without CO_2_. Under a microscope, the positive expression of β-galactosidase was observed under a light microscope to microscope and captured.

### Effects of retinol synergistic ferulic acid on HaCaT cell morphology and ROS

HaCaT cells were irradiated with UVB (56 mJ/ cm^2^) to induce cell senescence. The cells were pretreated with mixture of VA and FA (100μM: 80nM), NAC, VE for 24 h before irradiation. Incubated for 24 h, photograph the cell morphology and fluorescence. HaCaT cells are stained with 5-(and-6)-carboxy-2’,7’-dichlorodihydrofluorescein (DCFH-DA), incubated in the dark for 30 min, and washed 3 times with PBS. Under fluorescence microscopy and excited with blue light, the emission image of the cell can be observed. Green fluorescence represented the amount of intracellular ROS that were bound. The stronger the fluorescence, the higher the ROS content was. The level of cellular ROS production was determined applying Novo Quanteon flow cytometer (Agilent ACEA, America) according to the manufacturer’s protocol. Using the GraphPad Prism 9.0 software, an Ordinary One-Way ANOVA was performed to compare the differences between the groups. The post hoc test Dunnett’s t-test was employed to ascertain the significance of the experimental outcomes.

### Collection of test samples

HaCaT cells (50×10^5^ cells) were seeded in 6 cm cell culture dishes, and the model group and the drug administration group were exposed to UVB rays. After the cells were adhered, pretreated cells with mixture of VA and FA (100 μM:80 nM), NAC, VE for 24 h before irradiation. Then incubated for 24h, cells were collected by trypsinization, washed with ice-cold PBS. Aspirated the cell culture supernatant and added 100 μL of ripa lysate for each dish. Scraped off the cells in the dishes, centrifuge at 5000 rpm for 5 mins, removed the pellet, collected the supernatant, and stored it at -80° C together with the cell culture supernatant as test sample.

### Measurement of MDA, SOD, GSH, CAT

The level of Malondialdehyde (MDA) qualification was measured for the evaluation of lipid peroxidation rate on HaCaT cells according to the manufacturer’s protocol. Absorbance was measured at 532 nm to qualify MDA. Superoxide Dismutase (SOD) activity was measured on HaCaT cells according to the manufacturer’s protocol. Absorbance was measured at 450 nm, and the values were directly proportional to the SOD inhibition rate. Content of reduced glutathione (GSH) was measured at 412 nm for detecting the content of total glutathione. Catalase activity (CAT) was evaluated in measuring absorbance at OD 570 nm.

The experiment strictly adhered to the principle of complete randomization. Between-group comparisons were conducted using ANOVA analysis. All results are presented as mean ± standard deviation. The calculations were performed using GraphPad Prism software.

### RNA extraction

After treatments, HaCaT cells were detached using trypsin digestion. The supernatant was discarded after centrifugation. Then cells were washed with RNase-free PBS and centrifuged. For each sample, 1 mL of Trizol reagent was added, mixed thoroughly. Following the addition of 0.2 mL chloroform and thorough mixing, the mixture was centrifuged at 4° C. The upper aqueous phase was collected. Subsequently, 0.5 mL isopropanol was added, mixed well, and left to stand for 10 minutes. The precipitate was obtained after centrifugation, followed by centrifugation after washing with ice-cold 75% ethanol. Total RNA was obtained by dissolving the pellet in DEPC-treated (RNase-free) water.

### Validation of transcriptome data

According to the kit NEBNext® UltraTM RNA Library Prep Kit, libraries were established using total RNA greater than 1 μg. RNA purity was detected by NanoPhotometer spectrophotometer. Precise quantification of RNA concentration using Qubit2.0 Fluorometer was done. Diluted the library to 1.5 ng/μL, and detected RNA integrity with Agilent 2100 bioanalyzer.

### Sequencing data analysis

The image data obtained from the high-throughput sequencer for sequenced fragments were converted into sequence data (reads) through CASAVA base calling. To ensure the quality and reliability of data analysis, reads containing adapters, reads with N (where N represents undetermined base information), and low-quality reads (reads with Qphred ≤ 20, comprising over 50% of the total read length) were removed. Paired-end clean reads were aligned to the reference genome using HISAT2 v2.0.5. A spliced alignment database was generated based on the gene model annotation file. StringTie [42] was employed for novel gene prediction. The feature Counts tool was utilized to compute the read counts mapped to each gene. DESeq2 R software (version 1.16.1) was employed for differential expression analysis between two compared groups. The Benjamini and Hochberg method was applied to adjust the P-values. Corrected P-values along with |log2FC| were used as significance thresholds for differentially expressed genes. The clusterProfiler R software facilitated Gene Ontology (GO) enrichment analysis and differential gene analysis in KEGG pathways for the differentially expressed genes. R Studio software was utilized for generating heatmaps and volcano plots for analysis. The locally implemented GSEA analysis tool (http://www.broadinstitute.org/gsea/index.jsp) was used for Gene Set Enrichment Analysis of GO and KEGG datasets specific to the species. The RNA-seq data was uploaded to public database, GEO database (https://www.ncbi.nlm.nih.gov/), The BioProject is PRJNA1069325.

### Measurement of cyclooxygenase-2 (COX-2)

After 24h of UVB irradiation and sample treatment, cell lysates were collected from each dish. The concentration of COX-2 was analyzed from cell lysates using Human Cyclooxygenase-2 (COX-2) ELISA Kit in accordance with the manufacturer’s instructions.

### Data and materials availability

All the data could be obtained from the Supplementary Material and contacting the corresponding authors.

## Supplementary Material

Supplementary Table 1

Supplementary Table 2

Supplementary Table 3

Supplementary Table 4

Supplementary Table 5

Supplementary Table 6

Supplementary Table 7

Supplementary Table 8

Supplementary Table 9

## References

[1] Ansary TM, Hossain MR, Kamiya K, Komine M, Ohtsuki M. Inflammatory Molecules Associated with Ultraviolet Radiation-Mediated Skin Aging. Int J Mol Sci. 2021; 22:3974. 10.3390/ijms22083974 33921444 PMC8069861

[2] Karthikeyan R, Kanimozhi G, Prasad NR, Agilan B, Ganesan M, Srithar G. Alpha pinene modulates UVA-induced oxidative stress, DNA damage and apoptosis in human skin epidermal keratinocytes. Life Sci. 2018; 212:150–8. 10.1016/j.lfs.2018.10.004 30292828

[3] Gendrisch F, Esser PR, Schempp CM, Wölfle U. Luteolin as a modulator of skin aging and inflammation. Biofactors. 2021; 47:170–80. 10.1002/biof.1699 33368702

[4] Bang E, Kim DH, Chung HY. Protease-activated receptor 2 induces ROS-mediated inflammation through Akt-mediated NF-κB and FoxO6 modulation during skin photoaging. Redox Biol. 2021; 44:102022. 10.1016/j.redox.2021.102022 34082382 PMC8182111

[5] Lee MJ, Agrahari G, Kim HY, An EJ, Chun KH, Kang H, Kim YS, Bang CW, Tak LJ, Kim TY. Extracellular Superoxide Dismutase Prevents Skin Aging by Promoting Collagen Production through the Activation of AMPK and Nrf2/HO-1 Cascades. J Invest Dermatol. 2021; 141:2344–53.e7. 10.1016/j.jid.2021.02.757 33836179

[6] Qian H, Shan Y, Gong R, Lin D, Zhang M, Wang C, Wang L. Mechanism of action and therapeutic effects of oxidative stress and stem cell-based materials in skin aging: Current evidence and future perspectives. Front Bioeng Biotechnol. 2023; 10:1082403. 10.3389/fbioe.2022.1082403 36698629 PMC9868183

[7] He Y, Hu Y, Jiang X, Chen T, Ma Y, Wu S, Sun J, Jiao R, Li X, Deng L, Bai W. Cyanidin-3-O-glucoside inhibits the UVB-induced ROS/COX-2 pathway in HaCaT cells. J Photochem Photobiol B. 2017; 177:24–31. 10.1016/j.jphotobiol.2017.10.006 29031211

[8] Fuller B. Role of PGE-2 and Other Inflammatory Mediators in Skin Aging and Their Inhibition by Topical Natural Anti-Inflammatories. Cosmetics. 2019; 6. 10.3390/cosmetics6010006

[9] Subedi L, Lee TH, Wahedi HM, Baek SH, Kim SY. Resveratrol-Enriched Rice Attenuates UVB-ROS-Induced Skin Aging via Downregulation of Inflammatory Cascades. Oxid Med Cell Longev. 2017; 2017:8379539. 10.1155/2017/8379539 28900534 PMC5576414

[10] Romana-Souza B, Silva-Xavier W, Monte-Alto-Costa A. Topical retinol attenuates stress-induced ageing signs in human skin *ex vivo*, throughEGFR activation viaEGF, but notERK andAP-1 activation. Exp Dermatol. 2019; 28:906–13. 10.1111/exd.13675 29704879

[11] Dhaliwal S, Rybak I, Ellis SR, Notay M, Trivedi M, Burney W, Vaughn AR, Nguyen M, Reiter P, Bosanac S, Yan H, Foolad N, Sivamani RK. Prospective, randomized, double-blind assessment of topical bakuchiol and retinol for facial photoageing. Br J Dermatol. 2019; 180:289–96. 10.1111/bjd.16918 29947134

[12] Chien AL, Kim DJ, Cheng N, Shin J, Leung SG, Nelson AM, Zang J, Suh H, Rainer B, Wallis L, Okoye GA, Loss M, Kang S. Biomarkers of Tretinoin Precursors and Tretinoin Efficacy in Patients With Moderate to Severe Facial Photodamage: A Randomized Clinical Trial. JAMA Dermatol. 2022; 158:879–86. 10.1001/jamadermatol.2022.1891 35675051 PMC9178500

[13] Milosheska D, Roškar R. Use of Retinoids in Topical Antiaging Treatments: A Focused Review of Clinical Evidence for Conventional and Nanoformulations. Adv Ther. 2022; 39:5351–75. 10.1007/s12325-022-02319-7 36220974 PMC9618501

[14] Zasada M, Budzisz E, Erkiert-Polguj A. A Clinical Anti-Ageing Comparative Study of 0.3 and 0.5% Retinol Serums: A Clinically Controlled Trial. Skin Pharmacol Physiol. 2020; 33:102–16. 10.1159/000508168 32428912

[15] Mancuso C, Santangelo R. Ferulic acid: pharmacological and toxicological aspects. Food Chem Toxicol. 2014; 65:185–95. 10.1016/j.fct.2013.12.024 24373826

[16] Kim J, Kim J, Lee YI, Almurayshid A, Jung JY, Lee JH. Effect of a topical antioxidant serum containing vitamin C, vitamin E, and ferulic acid after Q-switched 1064-nm Nd:YAG laser for treatment of environment-induced skin pigmentation. J Cosmet Dermatol. 2020; 19:2576–82. 10.1111/jocd.13323 32052907

[17] Nogales C, Mamdouh ZM, List M, Kiel C, Casas AI, Schmidt HH. Network pharmacology: curing causal mechanisms instead of treating symptoms. Trends Pharmacol Sci. 2022; 43:136–50. 10.1016/j.tips.2021.11.004 34895945

[18] Acquaro M, Scelsi L, Pasotti B, Seganti A, Spolverini M, Greco A, Schirinzi S, Turco A, Sanzo A, Savastano S, Rordorf R, Ghio S. Sacubitril/valsartan effects on arrhythmias and left ventricular remodelling in heart failure: An observational study. Vascul Pharmacol. 2023; 152:107196. 10.1016/j.vph.2023.107196 37467909

[19] Wang Y, Yuan Y, Wang W, He Y, Zhong H, Zhou X, Chen Y, Cai XJ, Liu LQ. Mechanisms underlying the therapeutic effects of Qingfeiyin in treating acute lung injury based on GEO datasets, network pharmacology and molecular docking. Comput Biol Med. 2022; 145:105454. 10.1016/j.compbiomed.2022.105454 35367781

[20] Jiao W, Mi S, Sang Y, Jin Q, Chitrakar B, Wang X, Wang S. Integrated network pharmacology and cellular assay for the investigation of an anti-obesity effect of 6-shogaol. Food Chem. 2022; 374:131755. 10.1016/j.foodchem.2021.131755 34883426

[21] Zhang H, Zhang Y, Li Y, Wang Y, Yan S, Xu S, Deng Z, Yang X, Xie H, Li J. Bioinformatics and Network Pharmacology Identify the Therapeutic Role and Potential Mechanism of Melatonin in AD and Rosacea. Front Immunol. 2021; 12:756550. 10.3389/fimmu.2021.756550 34899707 PMC8657413

[22] Zhou W, Zhang H, Wang X, Kang J, Guo W, Zhou L, Liu H, Wang M, Jia R, Du X, Wang W, Zhang B, Li S. Network pharmacology to unveil the mechanism of Moluodan in the treatment of chronic atrophic gastritis. Phytomedicine. 2022; 95:153837. 10.1016/j.phymed.2021.153837 34883416

[23] Yan P, Wei Y, Wang M, Tao J, Ouyang H, Du Z, Li S, Jiang H. Network pharmacology combined with metabolomics and lipidomics to reveal the hypolipidemic mechanism of Alismatis rhizoma in hyperlipidemic mice. Food Funct. 2022; 13:4714–33. 10.1039/d1fo04386b 35383784

[24] Liou JY, Ting CK, Teng WN, Mandell MS, Tsou MY. Adaptation of non-linear mixed amount with zero amount response surface model for analysis of concentration-dependent synergism and safety with midazolam, alfentanil, and propofol sedation. Br J Anaesth. 2018; 120:1209–18. 10.1016/j.bja.2018.01.041 29793588

[25] Delage B, Bairras C, Buaud B, Pallet V, Cassand P. A high-fat diet generates alterations in nuclear receptor expression: prevention by vitamin A and links with cyclooxygenase-2 and beta-catenin. Int J Cancer. 2005; 116:839–46. 10.1002/ijc.21108 15856452

[26] Dieamant G, Pereda Mdel CV, Nogueira C, Eberlin S, Facchini G, Checon JT, Cesar CK, Mussi L, Polezel MA, Martins-Oliveira D Jr, Di Stasi LCD. Antiageing Mechanisms of a Standardized Supercritical CO 2 Preparation of Black Jack (Bidens pilosa L.) in Human Fibroblasts and Skin Fragments. Evid Based Complement Alternat Med. 2015; 2015:280529. 10.1155/2015/280529 25883669 PMC4391488

[27] Cruickshanks N, Zhang Y, Hine S, Gibert M, Yuan F, Oxford M, Grello C, Pahuski M, Dube C, Guessous F, Wang B, Deveau C, Saoud K, et al. Discovery and Therapeutic Exploitation of Mechanisms of Resistance to MET Inhibitors in Glioblastoma. Clin Cancer Res. 2019; 25:663–73. 10.1158/1078-0432.CCR-18-0926 30201763 PMC6335175

[28] Reed JR, Leon RP, Hall MK, Schwertfeger KL. Interleukin-1beta and fibroblast growth factor receptor 1 cooperate to induce cyclooxygenase-2 during early mammary tumourigenesis. Breast Cancer Res. 2009; 11:R21. 10.1186/bcr2246 19393083 PMC2688950

[29] Biran A, Zada L, Abou Karam P, Vadai E, Roitman L, Ovadya Y, Porat Z, Krizhanovsky V. Quantitative identification of senescent cells in aging and disease. Aging Cell. 2017; 16:661–71. 10.1111/acel.12592 28455874 PMC5506427

[30] Lowry WE. Its written all over your face: The molecular and physiological consequences of aging skin. Mech Ageing Dev. 2020; 190:111315. 10.1016/j.mad.2020.111315 32681843 PMC8911920

[31] Cocetta V, Cadau J, Saponaro M, Giacomini I, Dall'Acqua S, Sut S, Catanzaro D, Orso G, Miolo G, Menilli L, Pagetta A, Ragazzi E, Montopoli M. Further assessment of Salvia haenkei as an innovative strategy to counteract skin photo-aging and restore the barrier integrity. Aging (Albany NY). 2021; 13:89–103. 10.18632/aging.202464 33424011 PMC7835004

[32] Sreedhar A, Aguilera-Aguirre L, Singh KK. Mitochondria in skin health, aging, and disease. Cell Death Dis. 2020; 11:444. 10.1038/s41419-020-2649-z 32518230 PMC7283348

[33] Gu Y, Han J, Jiang C, Zhang Y. Biomarkers, oxidative stress and autophagy in skin aging. Ageing Res Rev. 2020; 59:101036. 10.1016/j.arr.2020.101036 32105850

[34] Lin JY, Kuo WW, Baskaran R, Kuo CH, Chen YA, Chen WS, Ho TJ, Day CH, Mahalakshmi B, Huang CY. Swimming exercise stimulates IGF1/ PI3K/Akt and AMPK/SIRT1/PGC1α survival signaling to suppress apoptosis and inflammation in aging hippocampus. Aging (Albany NY). 2020; 12:6852–64. 10.18632/aging.103046 32320382 PMC7202519

[35] Tudor DV, Bâldea I, Lupu M, Kacso T, Kutasi E, Hopârtean A, Stretea R, Gabriela Filip A. COX-2 as a potential biomarker and therapeutic target in melanoma. Cancer Biol Med. 2020; 17:20–31. 10.20892/j.issn.2095-3941.2019.0339 32296574 PMC7142851

[36] Guo H, Guo S, Liu H. Antioxidant activity and inhibition of ultraviolet radiation-induced skin damage of Selenium-rich peptide fraction from selenium-rich yeast protein hydrolysate. Bioorg Chem. 2020; 105:104431. 10.1016/j.bioorg.2020.104431 33161251

[37] Papaccio F, D Arino A, Caputo S, Bellei B. Focus on the Contribution of Oxidative Stress in Skin Aging. Antioxidants (Basel). 2022; 11:1121. 10.3390/antiox11061121 35740018 PMC9220264

[38] Nguyen JK, Mancebo S, Bleicher B, Jagdeo J. Successful Treatment of Porokeratosis With Ablative Fractional Carbon Dioxide Laser and Vitamin C, E, and Ferulic Acid Serum. J Drugs Dermatol. 2019; 18:174–6. 31741362

[39] Fang P, Han Y, Qu Y, Wang X, Zhang Y, Zhang W, Zhang N, Li G, Ma W. EIF3B stabilizes PTGS2 expression by counteracting MDM2-mediated ubiquitination to promote the development and progression of malignant melanoma. Cancer Sci. 2022; 113:4181–92. 10.1111/cas.15543 36050601 PMC9746036

[40] White DB, Slocum HK, Brun Y, Wrzosek C, Greco WR. A new nonlinear mixture response surface paradigm for the study of synergism: a three drug example. Curr Drug Metab. 2003; 4:399–409. 10.2174/1389200033489316 14529372

[41] Badawy MSEM, Elkhatib WF, Shebl RI. Mathematical pharmacodynamic modeling for antimicrobial assessment of ceftazidime/colistin versus gentamicin/meropenem combinations against carbapenem-resistant Pseudomonas aeruginosa biofilm. Ann Clin Microbiol Antimicrob. 2023; 22:53. 10.1186/s12941-023-00597-9 37394468 PMC10315054

[42] Pertea M, Pertea GM, Antonescu CM, Chang TC, Mendell JT, Salzberg SL. StringTie enables improved reconstruction of a transcriptome from RNA-seq reads. Nat Biotechnol. 2015; 33:290–5. 10.1038/nbt.3122 25690850 PMC4643835

